# Transient receptor potential melastatin 4 and cell death

**DOI:** 10.1007/s00424-012-1166-z

**Published:** 2012-10-13

**Authors:** J. Marc Simard, S. Kyoon Woo, Volodymyr Gerzanich

**Affiliations:** 1 Department of Neurosurgery, University of Maryland School of Medicine, 22 S. Greene Street, Suite S12D, Baltimore, MD 21201-1595 USA; 2 Department of Pathology, University of Maryland School of Medicine, Baltimore, MD USA; 3 Department of Physiology, University of Maryland School of Medicine, Baltimore, MD USA

**Keywords:** TRPM4, Necrosis, Apoptosis, Oncosis, Sodium, Depolarization, Review

## Abstract

Cell death proceeds by way of a variety of “cell death subroutines,” including several types of “apoptosis,” “regulated necrosis,” and others. “Accidental necrosis” due to profound adenosine triphosphate (ATP) depletion or oxidative stress is distinguished from regulated necrosis by the absence of death receptor signaling. However, both accidental and regulated necrosis have in common the process of “oncosis,” a physiological process characterized by Na^+^ influx and cell volume increase that, in necrotic cell death, is required to produce the characteristic features of membrane blebbing and membrane rupture. Here, we review emerging evidence that the monovalent cation channel, transient receptor potential melastatin 4 (TRPM4), is involved in the cell death process of oncosis. Potential involvement of TRPM4 in oncosis is suggested by the fact that the two principal regulators of TRPM4, intracellular ATP and Ca^2+^, are both altered during necrosis in the direction that causes TRPM4 channel opening. Under physiological conditions, activation of TRPM4 promotes Na^+^ influx and cell depolarization. Under pathological conditions, unchecked activation of TRPM4 leads to Na^+^ overload, cell volume increase, blebbing and cell membrane rupture, the latter constituting the irreversible end stage of necrosis. Emerging data indicate that TRPM4 plays a crucial role as end executioner in the accidental necrotic death of ATP-depleted or redox-challenged endothelial and epithelial cells, both in vitro and in vivo. Future studies will be needed to determine whether TRPM4 also plays a role in regulated necrosis and apoptosis.

## Introduction

Transient receptor potential (TRP) melastatin 4 (TRPM4) is a member of a large superfamily consisting of 28 mammalian cation channels. All but two TRP channels are permeable to divalent cations. The exceptions, TRPM4 and TRPM5, are non-selective, Ca^2+^-impermeable channels that transport monovalent cations exclusively [76]. TRPM4 and TRPM5 are both activated by increasing intracellular Ca^2+^. With TRPM4, ATP plays a crucial role in maintaining Ca^2+^ sensitivity through direct binding to the channel protein [77]. TRPM4, but not TRPM5, is blocked by intracellular ATP, i.e., is activated by decreasing intracellular ATP. Excellent reviews on the biophysical properties and physiological regulation of these channels have been published [40, 56, 59, 108, 110].

The best known function of TRPM4, the regulation of Ca^2+^ influx, is linked to one of the principal factors that regulates channel opening — the intracellular Ca^2+^ concentration [55, 56, 72, 77]. TRPM4 is activated following receptor-mediated Ca^2+^ mobilization, with activation causing depolarization of the cell membrane. Because the electrochemical driving force for Ca^2+^ is determined by the cell membrane potential, the reduction in membrane potential induced by activation of TRPM4 reduces the driving force for Ca^2+^ entry through Ca^2+^-permeable pathways. However, this mechanism for regulating Ca^2+^ entry may be dangerous, as it risks Na^+^ overload. As discussed below, Na^+^ overload plays a crucial role in cell death processes.

Surprisingly, the second major factor that regulates channel opening, the intracellular concentration of ATP, has a more obscure functional role. As noted above, ATP binding to the channel helps to maintaining Ca^2+^ sensitivity [77]. However, the functional role of channel block by intracellular ATP is uncertain. It has been speculated that this property confers sensitivity to the metabolic state of the cell [78], but whether this occurs under physiological conditions, and what its implications might be are unclear. The concentration of ATP that yields half-maximum open channel probability is <5 μM, far below the normal operating levels of 1–6 mM cytoplasmic ATP found in mammalian cells [10]. The only metabolic state associated with such levels of ATP is one of severe metabolic depletion bordering on cell death. This property of TRPM4 also may be dangerous, as it risks persistent channel opening if metabolic conditions are not rapidly improved and cellular levels of ATP are not adequately restored. Again, unchecked channel opening can lead to Na^+^ overload and its deleterious consequences, including cell death.

Despite its relatively recent discovery, much has been written about this unique ion channel. Excellent reviews of a general nature as well as specialized reviews focused on organ systems have been published [3, 17, 29, 32, 38, 42, 79, 91, 107]. There is growing recognition that TRPM4 plays a crucial role in a variety of diseases [74, 80]. Recent work has shown that mutations in the TRPM4 gene are responsible for certain cardiac conduction diseases [51, 60, 68, 93, 102]. In addition, TRPM4 plays a central role in cardiac hypertrophy [37, 39, 81], certain forms of hypertension [65], cutaneous anaphylaxis [32, 111], certain types of cancer [5, 57, 89], as well as spinal cord injury [35, 98]. However, one topic that has gained relatively little attention is the role of TRPM4 in cell death. Other transient receptor potential channels have been implicated in cell death, typically linked to Ca^2+^ influx [1, 2, 66, 67, 97]. Here, we review emerging data in which specific involvement of TRPM4 in accidental necrotic cell death has been shown, and we speculate on potential involvement in regulated necrosis and in apoptosis, which is theoretically possible but has yet to be demonstrated.

## Necrotic cell death

First, because of the variable usage in the nomenclature of cell death, it is appropriate to begin with a clarification of our usage of terms. Traditionally, different types of cell death were classified based on *morphological* features and included “apoptosis,” “necrosis” and “mitotic catastrophe” [50]. Currently, a *functional* classification of “cell death subroutines” is favored that is defined by a series of precise, measurable biochemical features, and includes “extrinsic apoptosis,” “caspase-dependent or -independent intrinsic apoptosis,” “regulated necrosis,” “autophagic cell death” and “mitotic catastrophe,” with these classifications applying both in vitro and in vivo [33, 34].

The current functional classification of cell death [34] is ambiguous as to whether necrosis in the context of severe ATP depletion or oxidative stress (absent death receptor signaling) should be termed “accidental necrosis” or “regulated necrosis.” Here, in keeping with tradition, we refer to it as accidental necrosis. The current classification does not include the term “oncosis” [34], which has been used by some authors to denote a form of necrotic cell death, i.e., necrotic death resulting from oncosis. Here, we use the term oncosis exclusively to refer to the physiological process of cell volume increase, in accord with the usage proffered by the Nomenclature Committee on Cell Death [50]. In this sense, oncosis is a process that is shared by both accidental and regulated necrosis. Thus, accidental necrosis can result either from oncosis (e.g., ATP depletion or oxidative stress) or from extremely harsh physical conditions (e.g., freeze–thaw cycles) [34].

Necrotic cells share specific morphological traits, including an increasingly translucent cytoplasm, the osmotic swelling of organelles, minor ultrastructural modifications of the nucleus (the dilatation of the nuclear membrane and the condensation of chromatin into small patches) and an increase in cell volume (oncosis), which culminates in the breakdown of the plasma membrane and loss of intracellular contents [33, 47, 50]. Necrotic cells do not fragment into discrete bodies, as their apoptotic counterparts do, nor do their nuclei, which may accumulate in necrotic tissues.

In necrosis, opening of the mitochondrial inner membrane permeability transition pore can cause irreversible mitochondrial inner membrane depolarization and osmotic mitochondrial lysis, impairing ATP formation and leading to massive energy depletion [49, 88, 90]. Mitochondrial swelling eventually ruptures the outer mitochondrial membrane, releasing intermembrane proteins. Other prominent features include formation of reactive oxygen species, activation of non-apoptotic proteases, and a large increase of intracellular Ca^2+^. Elevated Ca^2+^ activates Ca^2+^-dependent proteases, such as calpains [61, 62], and triggers mitochondrial Ca^2+^ overload, leading to further depolarization of the inner mitochondrial membrane and inhibition of ATP production.

**Fig. 1:**
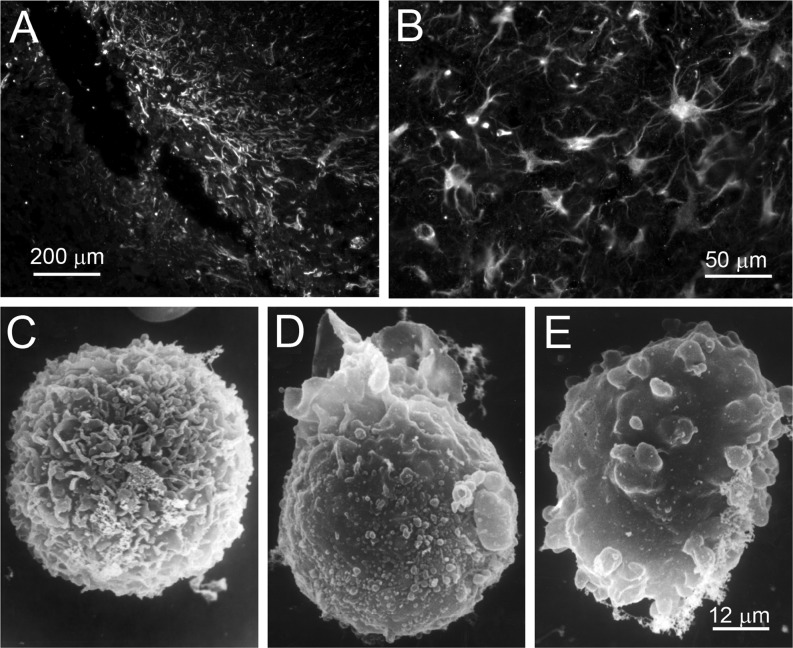
Cells expressing TRPM4 are highly susceptible to ATP-depletion-induced cell blebbing. **a**, **b** Immunolabeling for TRPM4 shows that native reactive astrocytes in situ that form a gliotic capsule surrounding a foreign body exhibit abundant expression of TRPM4 (Simard and colleagues, unpublished). **c**–**e** Scanning electron micrographs of freshly isolated native reactive astrocytes from a gliotic capsule showing that ATP depletion (1 mM sodium azide) induces oncotic blebbing; formaldehyde–glutaraldehyde fixed cells were imaged under control conditions (**c**), 5 min after exposure to sodium azide (**d**), and 25 min after exposure to sodium azide (**e**); bar, 12 μm; from Chen and Simard [24]

Absent direct physical destruction, accidental necrotic cell death, for example death due to severe ATP depletion or oxidative stress, requires that two events transpire: (1) the cytoskeleton first must become disrupted; (2) intracellular pressure must act to expand the cell volume (oncosis), resulting initially in blebbing and culminating in cell membrane rupture. Blebbing occurs when the cell membrane detaches from the cytoskeleton and is forced outward by intracellular pressure [106] (Fig. 1).

## ATP depletion

ATP depletion is a typical feature of necrosis. Initiation of necrosis generally requires that ATP levels be depleted by 80–85 % or more [50, 63]. ATP depletion due to factors external to the cell, e.g., following a traumatic insult or an ischemic event without reperfusion, results in accidental necrosis. The situation is more complex in the case of regulated necrosis. It is generally acknowledged that maintenance of ATP stores is required, at least initially, to pursue any form of programmed cell death, including regulated necrosis. Some evidence suggests that ATP-depletion may not be an absolute requirement for regulated necrosis [82]. However, in the type of regulated necrosis induced by tumor necrosis factor (TNF), which is called necroptosis, ATP-consuming processes including poly(ADP-ribose) polymerase-1 (PARP1) activity, translation and proteasome-mediated degradation persist and hence may contribute to the lethal decline in intracellular ATP [58, 109]. In addition, TNF induces receptor-interacting protein (RIP)-dependent inhibition of adenine nucleotide translocase (ANT)-mediated transport of ADP into mitochondria, which reduces ATP production and contributes further to the lethal decline in intracellular ATP [105]. In necroptosis induced by TNF-related apoptosis inducing ligand (TRAIL) at acidic extracellular pH, TRAIL gives rise to an early, 90 % depletion of intracellular ATP that is PARP-1-dependent [45]. Thus, in general, ATP depletion can be considered a characteristic feature of both accidental and regulated necrosis.

ATP depletion has striking effects on cytoskeletal structure and function. Disruption of actin filaments (F-actin) during ATP-depletion reflects predominantly the severing or fragmentation of F-actin [115], with depolymerization playing a contributory role [96]. Actin sequestration progresses in a duration-dependent manner, occurring as early as 15 min after onset of anoxia, when cellular ATP drops to <5 % of control levels [114]. Alterations in membrane–cytoskeleton linker proteins (spectrin, ankyrin, ezrin, myosin-1β and others) [73, 95, 113] induced by ATP depletion weaken membrane–cytoskeleton interactions, setting the stage for the later formation of blebs [22, 23, 70]. After 30 min of ATP depletion, the force required to pull the membrane away from the underlying cellular matrix diminishes by >95 %, which coincides with the time of bleb formation [27]. During ATP depletion, the strength of “membrane retention” forces diminishes until intracellular pressures become capable of initiating and driving membrane bleb formation.

Initially, as ATP-depleted cells swell and bleb, their plasma membranes remain “intact,” appearing to be under tension, yet becoming increasingly permeable to macromolecules [28]. As energy depletion proceeds, the plasma membrane becomes permeable to larger and larger molecules, a phenomenon that has been divided into three phases [22, 23]. In phases 1, 2, and 3, respectively, plasma membranes become permeable first to propidium iodide (PI; 668 Da), then to 3-kDa dextrans, and finally to 70-kDa dextrans or lactate dehydrogenase (140 kDa). Phase 1, which is marked by an increase in permeability to PI, is said to be reversible by reoxygenation [22, 106], an observation that would seem to conflict with the notion that PI uptake is a hallmark of necrotic cell death [50]. In any case, these observations on increasing permeability indicate that blebs do not actually have to rupture in order to begin the pre-morbid exchange of vital substances between the intracellular and extracellular compartments.

## Oncosis

Regulated and accidental forms of necrosis share several characteristic features. Not only is ATP depleted in both forms, but both also are characterized by cytoplasmic swelling (oncosis) and rupture of the plasma membrane [50]. Initially, cellular injury causes the formation of membrane blebs. Later, if the injurious stimulus persists, membrane blebs rupture and cell lysis occurs. Blebbing and membrane rupture are two essential features that characterize necrotic cell death [7, 47].

The loss of cytoskeletal support alone is not sufficient for anoxic plasma membrane disruption [21, 94]. In addition, an outward force is necessary to cause the cell to expand and for blebs to form. This outward force is provided by osmotic pressure, and it results in the process termed oncosis [26, 106]. The greater the osmotic pressure, the more rapidly blebs expand and rupture, resulting in frank irreversible disruption of the cell membrane.

One certain way to increase cellular osmotic pressure is to increase the influx of Na^+^ [20]. Indeed, necrosis has been said to require a combination of low ATP and high Na^+^ intracellularly [7]. Because Na^+^ is naturally excluded from the intracellular compartment, there normally exists a large electrochemical driving force for its passive inward transport. Increasing the influx of Na^+^ inevitably increases the inward driving force for Cl^–^, which helps to maintain intracellular electrical neutrality. The resulting increase in osmotically active Na^+^ and Cl^–^ ions intracellularly drives the influx of H_2_O, initiating cell swelling and culminating in membrane bleb formation.

One of several mechanisms involving altered function of active or passive ion transporters may give rise to the increase in intracellular Na^+^ that drives necrosis. Historically, it was thought that a key deleterious effect of ATP depletion was the loss in function of the active ion transporter, Na^+^–K^+^ ATPase, which normally extrudes Na^+^ from the cell. Loss of function of Na^+^–K^+^ ATPase results in a slow accumulation of Na^+^ intracellularly that is associated with slow depolarization. However, accumulating intracellular Na^+^ in this manner is not inevitably associated with an increase in intracellular pressure sufficient to produce necrosis. In energized cells, osmotic swelling induced by Na^+^–K^+^ ATPase inhibition with ouabain that is sufficient to cause a doubling of the cell volume does not produce blebbing or cell death [46]. Moreover, the effect of ouabain on cell death may be cell-specific. In some cells, the death signal is mediated by an interaction between ouabain and the Na^+^–K^+^ ATPase α-subunit but is independent of the inhibition of Na^+^–K^+^ pump-mediated ion fluxes and elevation of the [Na^+^]_i_/[K^+^]_i_ ratio [83, 84]. Overall, Na^+^–K^+^ ATPase inhibition may produce no death [85], only necrotic death [86], or a “mixed” form of death, with features of both necrosis and apoptosis in various cell types [83, 84, 87, 116, 118]. It is clear that, by itself, Na^+^–K^+^ ATPase inhibition is inadequate to account broadly for necrosis.

Alternatively, sodium influx may be augmented by opening a non-selective cation channel such as TRPM4. Pharmacological inhibition of non-selective cation channels using flufenamic acid abolishes cytosolic Ca^2+^ overload, cell swelling and necrosis of liver cells exposed to free-radical donors [8]. Implicating TRPM4 specifically in necrotic death makes theoretical sense, since the two principal regulators of TRPM4, intracellular ATP and Ca^2+^ [40, 59, 110], are both characteristically altered during necrosis and, moreover, are altered in the direction that causes TRPM4 channels to open: a decrease in intracellular ATP (see above) and an increase in intracellular Ca^2+^ [61, 62].

**Fig. 2:**
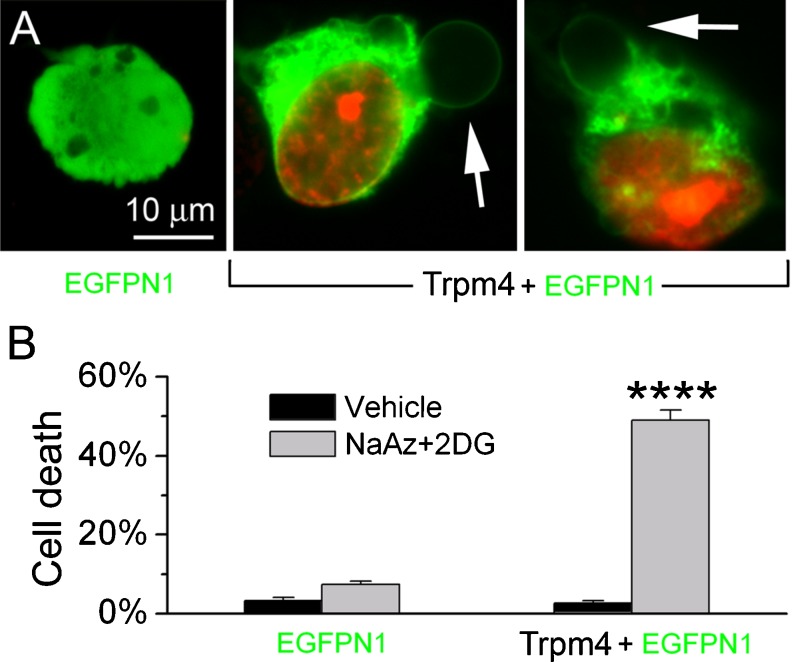
TRPM4 plays an obligate role in necrotic cell death in vitro. **a** Oncotic blebbing and nuclear labeling with propidium iodide (*PI*; *red*) induced by ATP depletion (1 mM sodium azide plus 10 mM 2-deoxyglucose [NaAz+2DG]) in COS-7 cells transfected with EGFPN1 + TRPM4 plasmid, but not in cells transfected with EGFPN1 plasmid alone. **b** Quantification of PI-positive necrotic cell death induced 10 min after ATP depletion in COS-7 cells transfected with EGFPN1 + TRPM4 plasmid or with EGFPN1 plasmid alone; values represent the percentage of the transfected cells (*green* cytoplasm) with nuclear PI labeling; experiments were performed in triplicate, with data from >100 cells per experiment; ****P < 0.0001; from Gerzanich et al. [35]

Involvement of TRPM4 in cell blebbing and necrotic cell death was shown first by Gerzanich et al. [35]. That this study involved accidental and not regulated necrosis was assured by the experimental design: COS-7 cells expressing TRPM4 were depleted rapidly of ATP, down to <2 % of control levels within 15 min, in the absence of TNFα or any other inducer of death receptor signaling. ATP depletion activated a 25-pS Cs^+^-permeable non-selective cation channel that was blocked by *N*-methyl-d-glucamine, characteristic of TRPM4. In COS-7 cells expressing TRPM4, ATP depletion caused marked cell blebbing, oncotic swelling and membrane leakage, and resulted in nuclear labeling by PI, consistent with necrotic cell death (Fig. 2).

Notably, in the study by Gerzanich et al. [35], ATP depletion did not induce necrotic death in COS-7 cells that did not express TRPM4. This finding is consistent with the observations above that the loss of cytoskeletal support or of Na^+^–K^+^ ATPase activity induced by ATP depletion is not sufficient to obtain plasma membrane disruption. Moreover, this finding indicates that in some cells, TRPM4 plays an obligate role as end executioner in necrotic cell death.

A distinct feature of heterologously expressed TRPM4 channels is that, upon activation by intracellular Ca^2+^, currents exhibit a fast decay due to a decrease in apparent sensitivity to Ca^2+^ [56, 75, 78]. This phenomenon could, in principal, act to protect cells from necrotic death by limiting Na^+^ influx. However, in HEK 293 cells expressing TRPM4, H_2_O_2_ was found to eliminate TRPM4 desensitization in a dose-dependent manner [99]. Site-directed mutagenesis revealed that the Cys^1093^ residue of TRPM4 is crucial for the H_2_O_2_-mediated reversal of desensitization. In the same study, it was shown that in HeLa cells, which endogenously express TRPM4, H_2_O_2_ (without ATP depletion) elicited necrosis as well as apoptosis, and that H_2_O_2_-mediated necrosis, but not apoptosis, was abolished by replacing external Na^+^ with *N*-methyl-d-glucamine or by knocking down TRPM4 with shRNA. Thus, removing TRPM4 desensitization by oxidative stress assures that TRPM4 will participate fully, without the impediment of desensitization, in the process of necrotic death.

TRPM4 recently was shown to be involved in the necrotic death of endothelial cells following exposure to lipopolysaccharide (LPS) [9]. Exposing human umbilical vein endothelial cells to LPS caused upregulation of TRPM4-like currents and caused Na^+^ overload, cell depolarization, cell volume increase and Na^+^-dependent necrotic cell death, as measured by release of lactate dehydrogenase. The cells were protected against LPS-induced necrotic death by 9-phenanthrol, a relatively selective inhibitor of TRPM4, by siRNA directed against TRPM4, as well as by suppression of TRPM4 using a dominant negative mutant.

**Fig. 3:**
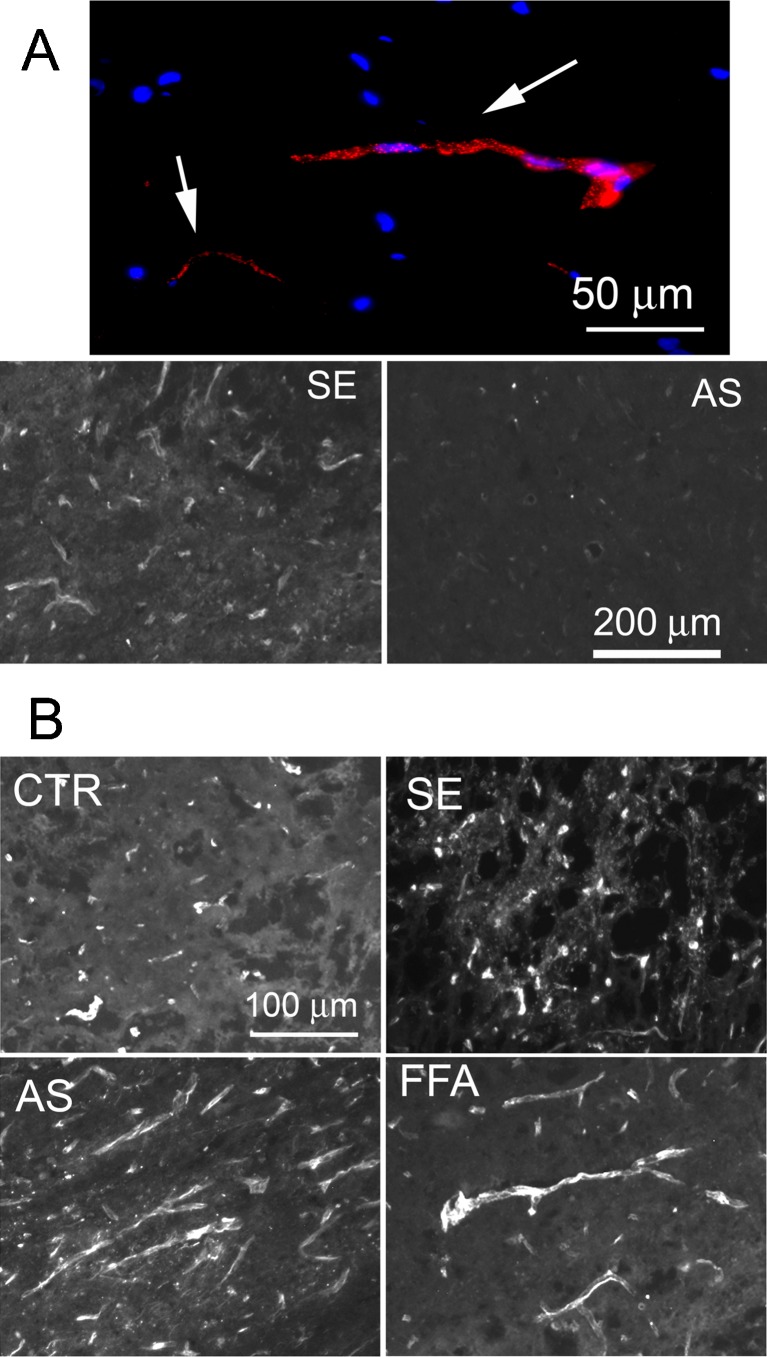
TRPM4 plays an obligate role in necrotic cell death in vivo. **a** *Upper panel:* fluorescence image of the penumbra 24 h after spinal cord injury (*SCI*) in a rat administered CY3-conjugated TRPM4 antisense (*AS*) oligodeoxynucleotide (*red*) by constant infusion post-SCI, showing that AS preferentially targets microvessels after SCI; rat perfused to remove intravascular contents; nuclei labeled with 4′,6-diamidino-2-phenylindole (*DAPI*; *blue*); *arrows* point to capillaries; *lower panel:* immunohistochemistry for TRPM4 in tissues obtained 24 h post-SCI from rats administered TRPM4-sense (*SE*) or TRPM4-AS, showing reduced TRPM4 expression with AS. **b** Spinal cord sections from an untreated control rat (*CTR*) and rats administered TRPM4-SE, TRPM4-AS or flufenamic acid (*FFA*), showing necrosis-induced capillary fragmentation in the controls (CTR and SE) and preservation of intact capillaries with gene suppression or pharmacological block of TRPM4; from Gerzanich et al. [35]

TRPM4 is involved in necrotic death in vivo as well, as shown first by Gerzanich et al. [35]. In this study, traumatic injury to the spinal cord was accompanied by delayed capillary fragmentation, resulting in the autodestructive process termed “progressive hemorrhagic necrosis.” Microvessels in the penumbra of injury showed prominent upregulation of TRPM4 mRNA and protein, which was not present in tissues remote from the injury. Capillary fragmentation was attributed to necrotic death of microvascular endothelial cells (Fig. 3). *TRPM4*−/− mice were completely spared from capillary fragmentation and progressive hemorrhagic necrosis. Moreover, rats that were subjected to a similar traumatic insult and that were administered antisense oligodeoxynucleotide directed against *TRPM4* also were spared from capillary fragmentation and progressive hemorrhagic necrosis. The latter series of experiments also showed that antisense entered microvascular endothelial cells in the penumbra almost exclusively, and thereby prevented the destruction (fragmentation) of microvessels (Fig. 3). Together, these findings are consistent with TRPM4 playing an obligate role as end executioner in necrotic cell death in vivo.

TRPM4 is not the only ion channel that can transport Na^+^ in a manner sufficient to cause oncosis in the context of ATP depletion. Many other ion channels passively transport Na^+^ down its electrochemical gradient, either selectively, e.g., voltage dependent Na^+^ channels, or non-selectively, e.g., non-selective cation channels such as *N*-methyl-d-aspartate (NMDA) receptor channels and some TRP channels, some of which transport both monovalent and divalent cations. However, with many non-selective cation channels including NMDA and many TRP, distinguishing between effects due to Na^+^ transport versus Ca^2+^ overload is difficult, and indeed, pronecrotic effects of these channels are typically attributed to Ca^2+^ influx. Other examples abound wherein Na^+^ influx is induced and is associated with oncosis and necrotic cell death, including such activators as venom from the wasp, *Nasonia vitripennis* [92] and a cytotoxic antibody that kills undifferentiated human embryonic stem cells [104]. However, none of these channels exhibits the unique combination of properties seen with TRPM4 of being activated by a decrease in ATP and an increase in Ca^2+^. Additional work will be required to determine the role of many of these channels specifically in passive Na^+^ transport that is requisite for necrotic cell death.

## Apoptotic cell death

Unlike accidental necrosis, apoptosis is a metabolically active, energy demanding process that maintains cellular ATP levels and plasma membrane integrity until late in the cell death process. Comprehensive reviews on the molecular machinery involved in apoptosis have been published. Here, we focus on the role of Na^+^ influx and the potential involvement of TRPM4.

Like necrosis, apoptotic cell death has features of Na^+^ dependence and cell membrane depolarization [12–15, 31, 87]. A variety of apoptotic stimuli result in an early transient increase in intracellular Na^+^ that is associated with marked plasma membrane depolarization that occurs prior to and after cell shrinkage [15]. In thymocytes, Na^+^ influx plays a major role in the rapid phosphatidylserine exposure induced by P2X7 receptor activation [25]. In Jurkat cells, inhibition of Na^+^ influx by ion substitution reduces Fas-induced apoptosis [13]. An initial Na^+^ influx is necessary for cell shrinkage, but not for the activation of the cell death effectors, whereas K^+^ efflux is critical for cell shrinkage and death by apoptosis. Downstream mechanisms activated by the rise in Na^+^ are not completely elucidated, but may include activation of a Na^+^–Ca^2+^ exchanger, resulting in Ca^+^ overload [11, 54, 69]. In addition, Na^+^ overload may be involved in opening of the mitochondrial inner membrane permeability transition pore and mitochondrial swelling, resulting in cytochrome *c* release and activation of the caspase-3-dependent apoptosis [30].

Several mechanisms have been postulated to account for the early rise of intracellular Na^+^ in apoptosis, including diminished function of Na^+^–K^+^ ATPase, augmented function of voltage-dependent Na^+^ channels, and augmented function of non-selective cation channels (see review by Franco et al. [31]). In general, changes in Na^+^ and K^+^ fluxes typical of apoptosis are likely to be caused by a complex interplay of several mechanisms, including a decrease in Na^+^–K^+^ ATPase activity, Na^+^–Cl^−^ co-transport and an increase in Na^+^ channel permeability [112].

Reflecting on the potential involvement of voltage-dependent Na^+^ channels is instructive. Unlike Na^+^–K^+^ ATPase and non-selective cation channels, voltage-dependent Na^+^ channels are highly selective passive transporters of Na^+^, leaving little doubt about the event that triggers apoptosis. Activation of voltage-dependent Na^+^ channels during oxygen deprivation leads to apoptotic neuronal death that is reduced by the highly specific Na^+^ channel blocker, tetrodotoxin [6]. Veratridine, which prevents inactivation of voltage-dependent Na^+^ channels, increases influx of Na^+^, causes cell depolarization, and induces apoptosis of neuronal cells [19, 36, 44, 117]. Following global cerebral ischemia in the gerbil, administration of the Na^+^ ionophore, monensin, or of the Na^+^ channel blocker, tetrodotoxin, results in an increase or a decrease, respectively, in apoptotic neuronal death in the hippocampus [16]. A gain-of-function mutation [the N(1325)S mutation] in the cardiac Na^+^ channel gene SCN5A results in an increase in apoptotic cell death of ventricular myoctes [119]. Such studies demonstrate the crucial role played by an early rise in Na^+^ in the cell death subroutine of apoptosis.

In some cases, a non-selective cation channel such as TRPM4 may be responsible for the early rise in intracellular Na^+^ involved in apoptosis. The involvement of non-selective cation channels in apoptosis has been widely reported in many cell types following exposure to various apoptotic stimuli [41, 43, 48, 52, 53, 64, 71, 101, 103]. However, most of the studies on non-selective cation channels attributed cell death signaling to a rise in intracellular Ca^2+^, with little consideration for the potential role of intracellular Na^+^ or of cell membrane potential.

A number of cells have been found to express a channel with properties of TRPM4 that could mediate an early rise in Na^+^ that may trigger apoptosis. H_2_O_2_, an inducer of apoptosis in epithelial cells [4, 18], increases the activity of a 24 pS Ca^2+^-activated, non-selective cation channel in a bronchial epithelial cell line [43], and of a 19 pS Ca^2+^-activated, ATP-sensitive non-selective cation channel in a liver-derived epithelial cell line [100]. Both of these studies are reminiscent of the effect of H_2_O_2_ on TRPM4 in endothelial cells [99]. Conversely, H_2_O_2_-induced apoptosis in HeLa cells, which express TRPM4, is not blocked by inhibiting Na^+^ influx with ion substitution [99]. Despite theoretical data pointing to a potential role of TRPM4 in triggering apoptosis, to our knowledge, there has been no molecular demonstration of this to date.

## Summary

Cell death is extraordinarily complex, with new molecular insights continuing to emerge at a rapid pace. The molecular events involved in apoptosis have been extensively studied, but by comparison, the molecular basis for necrosis is less well understood. Much progress has been realized during the last decade, not the least important of which is the recognition that necrosis may proceed by accidental as well as by regulated pathways, with both requiring Na^+^ influx to drive oncosis that is responsible for membrane blebbing and rupture. A variety of TRP channels have been implicated in apoptotic and necrotic cell death, typically related to Ca^2+^ influx [1, 2, 66, 67, 97]. Emerging evidence indicates that the monovalent cation channel, TRPM4, which under physiological conditions promotes Na^+^ influx and cell depolarization, plays a crucial role as end executioner in the accidental necrotic death of ATP-depleted or redox-challenged endothelial and epithelial cells, both in vitro and in vivo. TRPM4 may also play a role in regulated necrosis and apoptosis, although future studies will be required to elucidate this.

## References

[1] Aarts MM, Tymianski M (2005). TRPM7 and ischemic CNS injury. Neuroscientist.

[2] Aarts MM, Tymianski M (2005). TRPMs and neuronal cell death. Pflugers Arch.

[3] Abriel H, Syam N, Sottas V, Amarouch MY, Rougier JS (2012). TRPM4 channels in the cardiovascular system: physiology, pathophysiology, and pharmacology. Biochem Pharmacol.

[4] Arends B, Slump E, Spee B, Rothuizen J, Penning LC (2008). Hepatocyte growth factor improves viability after H_2_O_2_-induced toxicity in bile duct epithelial cells. Comp Biochem Physiol C Toxicol Pharmacol.

[5] Armisen R, Marcelain K, Simon F, Tapia JC, Toro J, Quest AF, Stutzin A (2011). TRPM4 enhances cell proliferation through up-regulation of the beta-catenin signaling pathway. J Cell Physiol.

[6] Banasiak KJ, Burenkova O, Haddad GG (2004). Activation of voltage-sensitive sodium channels during oxygen deprivation leads to apoptotic neuronal death. Neuroscience.

[7] Barros LF, Hermosilla T, Castro J (2001). Necrotic volume increase and the early physiology of necrosis. Comp Biochem Physiol A Mol Integr Physiol.

[8] Barros LF, Stutzin A, Calixto A, Catalan M, Castro J, Hetz C, Hermosilla T (2001). Nonselective cation channels as effectors of free radical-induced rat liver cell necrosis. Hepatology.

[9] Becerra A, Echeverria C, Varela D, Sarmiento D, Armisen R, Nunez-Villena F, Montecinos M, Simon F (2011). Transient receptor potential melastatin 4 inhibition prevents lipopolysaccharide-induced endothelial cell death. Cardiovasc Res.

[10] Beis I, Newsholme EA (1975). The contents of adenine nucleotides, phosphagens and some glycolytic intermediates in resting muscles from vertebrates and invertebrates. Biochem J.

[11] Blaustein MP, Lederer WJ (1999). Sodium/calcium exchange: its physiological implications. Physiol Rev.

[12] Bortner CD, Cidlowski JA (2002). Apoptotic volume decrease and the incredible shrinking cell. Cell Death Differ.

[13] Bortner CD, Cidlowski JA (2003). Uncoupling cell shrinkage from apoptosis reveals that Na + influx is required for volume loss during programmed cell death. J Biol Chem.

[14] Bortner CD, Cidlowski JA (2007). Cell shrinkage and monovalent cation fluxes: role in apoptosis. Arch Biochem Biophys.

[15] Bortner CD, Gomez-Angelats M, Cidlowski JA (2001). Plasma membrane depolarization without repolarization is an early molecular event in anti-Fas-induced apoptosis. J Biol Chem.

[16] Brahma MK, Dohare P, Varma S, Rath SK, Garg P, Biswal PK, Chowdhury PD, Ray M (2009). The neuronal apoptotic death in global cerebral ischemia in gerbil: important role for sodium channel modulator. J Neurosci Res.

[17] Brayden JE, Earley S, Nelson MT, Reading S (2008). Transient receptor potential (TRP) channels, vascular tone and autoregulation of cerebral blood flow. Clin Exp Pharmacol Physiol.

[18] Bucchieri F, Puddicombe SM, Lordan JL, Richter A, Buchanan D, Wilson SJ, Ward J, Zummo G, Howarth PH, Djukanovic R, Holgate ST, Davies DE (2002). Asthmatic bronchial epithelium is more susceptible to oxidant-induced apoptosis. Am J Respir Cell Mol Biol.

[19] Callaway JK, Beart PM, Jarrott B, Giardina SF (2001). Incorporation of sodium channel blocking and free radical scavenging activities into a single drug, AM-36, results in profound inhibition of neuronal apoptosis. Br J Pharmacol.

[20] Carini R, Autelli R, Bellomo G, Albano E (1999). Alterations of cell volume regulation in the development of hepatocyte necrosis. Exp Cell Res.

[21] Chen J, Dai J, Grant RL, Doctor RB, Sheetz MP, Mandel LJ (1997). Loss of cytoskeletal support is not sufficient for anoxic plasma membrane disruption in renal cells. Am J Physiol.

[22] Chen J, Liu X, Mandel LJ, Schnellmann RG (2001). Progressive disruption of the plasma membrane during renal proximal tubule cellular injury. Toxicol Appl Pharmacol.

[23] Chen J, Wagner MC (2001). Altered membrane–cytoskeleton linkage and membrane blebbing in energy-depleted renal proximal tubular cells. Am J Physiol Renal Physiol.

[24] Chen M, Simard JM (2001). Cell swelling and a nonselective cation channel regulated by internal Ca^2+^ and ATP in native reactive astrocytes from adult rat brain. J Neurosci.

[25] Courageot MP, Lepine S, Hours M, Giraud F, Sulpice JC (2004). Involvement of sodium in early phosphatidylserine exposure and phospholipid scrambling induced by P2X7 purinoceptor activation in thymocytes. J Biol Chem.

[26] Dai J, Sheetz MP (1999). Membrane tether formation from blebbing cells. Biophys J.

[27] Doctor RB, Zhelev DV, Mandel LJ (1997). Loss of plasma membrane structural support in ATP-depleted renal epithelia. Am J Physiol.

[28] Dong Z, Patel Y, Saikumar P, Weinberg JM, Venkatachalam MA (1998). Development of porous defects in plasma membranes of adenosine triphosphate-depleted Madin–Darby canine kidney cells and its inhibition by glycine. Lab Invest.

[29] Earley S, Reading S, Brayden JE (2007) Functional significance of transient receptor potential channels in vascular function. In: Liedtke WB, Heller S (eds) TRP ion channel function in sensory transduction and cellular signaling cascades, chapter 26. CRC Press, Boca Raton. http://www.crcpress.com/. Accessed 5 Sept 2012

[30] Fang KM, Lee AS, Su MJ, Lin CL, Chien CL, Wu ML (2008). Free fatty acids act as endogenous ionophores, resulting in Na^+^ and Ca^2+^ influx and myocyte apoptosis. Cardiovasc Res.

[31] Franco R, Bortner CD, Cidlowski JA (2006). Potential roles of electrogenic ion transport and plasma membrane depolarization in apoptosis. J Membr Biol.

[32] Freichel M, Almering J, Tsvilovskyy V (2012). The role of TRP proteins in mast cells. Front Immunol.

[33] Galluzzi L, Vanden Berghe T, Vanlangenakker N, Buettner S, Eisenberg T, Vandenabeele P, Madeo F, Kroemer G (2011). Programmed necrosis from molecules to health and disease. Int Rev Cell Mol Biol.

[34] Galluzzi L, Vitale I, Abrams JM, Alnemri ES, Baehrecke EH, Blagosklonny MV, Dawson TM, Dawson VL, El-Deiry WS, Fulda S, Gottlieb E, Green DR, Hengartner MO, Kepp O, Knight RA, Kumar S, Lipton SA, Lu X, Madeo F, Malorni W, Mehlen P, Nunez G, Peter ME, Piacentini M, Rubinsztein DC, Shi Y, Simon HU, Vandenabeele P, White E, Yuan J, Zhivotovsky B, Melino G, Kroemer G (2012). Molecular definitions of cell death subroutines: recommendations of the Nomenclature Committee on Cell Death 2012. Cell Death Differ.

[35] Gerzanich V, Woo SK, Vennekens R, Tsymbalyuk O, Ivanova S, Ivanov A, Geng Z, Chen Z, Nilius B, Flockerzi V, Freichel M, Simard JM (2009). De novo expression of Trpm4 initiates secondary hemorrhage in spinal cord injury. Nat Med.

[36] Gomez-Lazaro M, Galindo MF, Fernandez-Gomez FJ, Prehn JH, Jordan J (2005). Activation of p53 and the pro-apoptotic p53 target gene PUMA during depolarization-induced apoptosis of chromaffin cells. Exp Neurol.

[37] Guinamard R, Bois P (2007). Involvement of transient receptor potential proteins in cardiac hypertrophy. Biochim Biophys Acta.

[38] Guinamard R, Demion M, Launay P (2010). Physiological roles of the TRPM4 channel extracted from background currents. Physiology (Bethesda).

[39] Guinamard R, Demion M, Magaud C, Potreau D, Bois P (2006). Functional expression of the TRPM4 cationic current in ventricular cardiomyocytes from spontaneously hypertensive rats. Hypertension.

[40] Guinamard R, Salle L, Simard C (2011). The non-selective monovalent cationic channels TRPM4 and TRPM5. Adv Exp Med Biol.

[41] Gutierrez AA, Arias JM, Garcia L, Mas-Oliva J, Guerrero-Hernandez A (1999). Activation of a Ca2 + -permeable cation channel by two different inducers of apoptosis in a human prostatic cancer cell line. J Physiol.

[42] Islam MS (2011). TRP channels of islets. Adv Exp Med Biol.

[43] Jeulin C, Dazy AC, Marano F (2002). Effects of hydrogen peroxide and hydroxyl radicals on the cytosolic side of a non-selective cation channel in the cultured human bronchial epithelial cell line 16HBE14o-. Pflugers Arch.

[44] Jordan J, Galindo MF, Calvo S, Gonzalez-Garcia C, Cena V (2000). Veratridine induces apoptotic death in bovine chromaffin cells through superoxide production. Br J Pharmacol.

[45] Jouan-Lanhouet S, Arshad MI, Piquet-Pellorce C, Martin-Chouly C, Le Moigne-Muller G, Van HF, Takahashi N, Sergent O, Lagadic-Gossmann D, Vandenabeele P, Samson M, Dimanche-Boitrel MT (2012) TRAIL induces necroptosis involving RIPK1/RIPK3-dependent PARP-1 activation. Cell Death Differ. doi:10.1038/cdd.2012.90 10.1038/cdd.2012.90 PMC3504714 22814620

[46] Jurkowitz-Alexander MS, Altschuld RA, Hohl CM, Johnson JD, McDonald JS, Simmons TD, Horrocks LA (1992). Cell swelling, blebbing, and death are dependent on ATP depletion and independent of calcium during chemical hypoxia in a glial cell line (ROC-1). J Neurochem.

[47] Kerr JF, Wyllie AH, Currie AR (1972). Apoptosis: a basic biological phenomenon with wide-ranging implications in tissue kinetics. Br J Cancer.

[48] Kim JA, Kang YS, Jung MW, Lee SH, Lee YS (1999). Involvement of Ca^2+^ influx in the mechanism of tamoxifen-induced apoptosis in HepG2 human hepatoblastoma cells. Cancer Lett.

[49] Kinnally KW, Peixoto PM, Ryu SY, Dejean LM (2011). Is mPTP the gatekeeper for necrosis, apoptosis, or both?. Biochim Biophys Acta.

[50] Kroemer G, Galluzzi L, Vandenabeele P, Abrams J, Alnemri ES, Baehrecke EH, Blagosklonny MV, El-Deiry WS, Golstein P, Green DR, Hengartner M, Knight RA, Kumar S, Lipton SA, Malorni W, Nunez G, Peter ME, Tschopp J, Yuan J, Piacentini M, Zhivotovsky B, Melino G (2009). Classification of cell death: recommendations of the Nomenclature Committee on Cell Death 2009. Cell Death Differ.

[51] Kruse M, Schulze-Bahr E, Corfield V, Beckmann A, Stallmeyer B, Kurtbay G, Ohmert I, Schulze-Bahr E, Brink P, Pongs O (2009). Impaired endocytosis of the ion channel TRPM4 is associated with human progressive familial heart block type I. J Clin Invest.

[52] Lang F, Birka C, Myssina S, Lang KS, Lang PA, Tanneur V, Duranton C, Wieder T, Huber SM (2004). Erythrocyte ion channels in regulation of apoptosis. Adv Exp Med Biol.

[53] Lang F, Lang KS, Wieder T, Myssina S, Birka C, Lang PA, Kaiser S, Kempe D, Duranton C, Huber SM (2003). Cation channels, cell volume and the death of an erythrocyte. Pflugers Arch.

[54] Lang F, Ritter M, Gamper N, Huber S, Fillon S, Tanneur V, Lepple-Wienhues A, Szabo I, Gulbins E (2000). Cell volume in the regulation of cell proliferation and apoptotic cell death. Cell Physiol Biochem.

[55] Launay P, Cheng H, Srivatsan S, Penner R, Fleig A, Kinet JP (2004). TRPM4 regulates calcium oscillations after T cell activation. Science.

[56] Launay P, Fleig A, Perraud AL, Scharenberg AM, Penner R, Kinet JP (2002). TRPM4 is a Ca^2+^-activated nonselective cation channel mediating cell membrane depolarization. Cell.

[57] Lehen'kyi V, Prevarskaya N (2011). Oncogenic TRP channels. Adv Exp Med Biol.

[58] Leist M, Single B, Castoldi AF, Kuhnle S, Nicotera P (1997). Intracellular adenosine triphosphate (ATP) concentration: a switch in the decision between apoptosis and necrosis. J Exp Med.

[59] Liman ER (2007) The Ca^2+^-activated TRP channels: TRPM4 and TRPM5. In: Liedtke WB, Heller S (eds) TRP ion channel function in sensory transduction and cellular signaling cascades, chapter 15. CRC Press, Boca Raton. http://www.crcpress.com/. Accessed 5 Sept 2012 21204486

[60] Liu H, El ZL, Kruse M, Guinamard R, Beckmann A, Bozio A, Kurtbay G, Megarbane A, Ohmert I, Blaysat G, Villain E, Pongs O, Bouvagnet P (2010). Gain-of-function mutations in TRPM4 cause autosomal dominant isolated cardiac conduction disease. Circ Cardiovasc Genet.

[61] Liu X, Schnellmann RG (2003). Calpain mediates progressive plasma membrane permeability and proteolysis of cytoskeleton-associated paxillin, talin, and vinculin during renal cell death. J Pharmacol Exp Ther.

[62] Liu X, Van VT, Schnellmann RG (2004). The role of calpain in oncotic cell death. Annu Rev Pharmacol Toxicol.

[63] Los M, Mozoluk M, Ferrari D, Stepczynska A, Stroh C, Renz A, Herceg Z, Wang ZQ, Schulze-Osthoff K (2002). Activation and caspase-mediated inhibition of PARP: a molecular switch between fibroblast necrosis and apoptosis in death receptor signaling. Mol Biol Cell.

[64] Manion MK, Su Z, Villain M, Blalock JE (2000). A new type of Ca(2+) channel blocker that targets Ca(2+) sensors and prevents Ca(2+)-mediated apoptosis. FASEB J.

[65] Mathar I, Vennekens R, Meissner M, Kees F, Van der Mieren G, Camacho Londono JE, Uhl S, Voets T, Hummel B, van den Bergh A, Herijgers P, Nilius B, Flockerzi V, Schweda F, Freichel M (2010). Increased catecholamine secretion contributes to hypertension in TRPM4-deficient mice. J Clin Invest.

[66] McNulty S, Fonfria E (2005). The role of TRPM channels in cell death. Pflugers Arch.

[67] Miller BA (2006). The role of TRP channels in oxidative stress-induced cell death. J Membr Biol.

[68] Mills RW, Milan DJ (2010). TRPM4-linked isolated cardiac conduction defects: bad trafficking causes electrical gridlock. Circ Cardiovasc Genet.

[69] Miyamoto S, Howes AL, Adams JW, Dorn GW, Brown JH (2005). Ca^2+^ dysregulation induces mitochondrial depolarization and apoptosis: role of Na^+^/Ca^2+^ exchanger and AKT. J Biol Chem.

[70] Molitoris BA, Dahl R, Hosford M (1996). Cellular ATP depletion induces disruption of the spectrin cytoskeletal network. Am J Physiol.

[71] Mukherjee SB, Das M, Sudhandiran G, Shaha C (2002). Increase in cytosolic Ca^2+^ levels through the activation of non-selective cation channels induced by oxidative stress causes mitochondrial depolarization leading to apoptosis-like death in Leishmania donovani promastigotes. J Biol Chem.

[72] Murakami M, Xu F, Miyoshi I, Sato E, Ono K, Iijima T (2003). Identification and characterization of the murine TRPM4 channel. Biochem Biophys Res Commun.

[73] Neisch AL, Fehon RG (2011). Ezrin, Radixin and Moesin: key regulators of membrane-cortex interactions and signaling. Curr Opin Cell Biol.

[74] Nilius B, Owsianik G, Voets T, Peters JA (2007). Transient receptor potential cation channels in disease. Physiol Rev.

[75] Nilius B, Prenen J, Droogmans G, Voets T, Vennekens R, Freichel M, Wissenbach U, Flockerzi V (2003). Voltage dependence of the Ca^2+^-activated cation channel TRPM4. J Biol Chem.

[76] Nilius B, Prenen J, Janssens A, Owsianik G, Wang C, Zhu MX, Voets T (2005). The selectivity filter of the cation channel TRPM4. J Biol Chem.

[77] Nilius B, Prenen J, Tang J, Wang C, Owsianik G, Janssens A, Voets T, Zhu MX (2005). Regulation of the Ca^2+^ sensitivity of the nonselective cation channel TRPM4. J Biol Chem.

[78] Nilius B, Prenen J, Voets T, Droogmans G (2004). Intracellular nucleotides and polyamines inhibit the Ca^2+^-activated cation channel TRPM4b. Pflugers Arch.

[79] Nilius B, Vennekens R (2006). From cardiac cation channels to the molecular dissection of the transient receptor potential channel TRPM4. Pflugers Arch.

[80] Nilius B, Voets T, Peters J (2005) TRP channels in disease. Sci STKE 2005:re8 10.1126/stke.2952005re8 16077087

[81] Nishida M, Kurose H (2008). Roles of TRP channels in the development of cardiac hypertrophy. Naunyn Schmiedebergs Arch Pharmacol.

[82] Ono K, Wang X, Kim SO, Armstrong LC, Bornstein P, Han J (2010). Metaxin deficiency alters mitochondrial membrane permeability and leads to resistance to TNF-induced cell killing. Protein Cell.

[83] Orlov SN, Hamet P (2004). Apoptosis vs. oncosis: role of cell volume and intracellular monovalent cations. Adv Exp Med Biol.

[84] Orlov SN, Hamet P (2006). The death of cardiotonic steroid-treated cells: evidence of Na + i, K + i-independent H + i-sensitive signalling. Acta Physiol (Oxf).

[85] Orlov SN, Thorin-Trescases N, Kotelevtsev SV, Tremblay J, Hamet P (1999). Inversion of the intracellular Na^+^/K^+^ ratio blocks apoptosis in vascular smooth muscle at a site upstream of caspase-3. J Biol Chem.

[86] Orlov SN, Thorin-Trescases N, Pchejetski D, Taurin S, Farhat N, Tremblay J, Thorin E, Hamet P (2004). Na^+^/K^+^ pump and endothelial cell survival: [Na^+^]_i_/[K^+^]_i_-independent necrosis triggered by ouabain, and protection against apoptosis mediated by elevation of [Na^+^]_i_. Pflugers Arch.

[87] Panayiotidis MI, Bortner CD, Cidlowski JA (2006). On the mechanism of ionic regulation of apoptosis: would the Na^+^/K^+^-ATPase please stand up?. Acta Physiol (Oxf).

[88] Pivovarova NB, Andrews SB (2010). Calcium-dependent mitochondrial function and dysfunction in neurons. FEBS J.

[89] Prevarskaya N, Zhang L, Barritt G (2007). TRP channels in cancer. Biochim Biophys Acta.

[90] Rasola A, Bernardi P (2011). Mitochondrial permeability transition in Ca(2+)-dependent apoptosis and necrosis. Cell Calcium.

[91] Reading SA, Brayden JE (2007). Central role of TRPM4 channels in cerebral blood flow regulation. Stroke.

[92] Rivers DB, Rocco MM, Frayha AR (2002). Venom from the ectoparasitic wasp *Nasonia vitripennis* increases Na^+^ influx and activates phospholipase C and phospholipase A2 dependent signal transduction pathways in cultured insect cells. Toxicon.

[93] Robertson GA (2009). Endocytic control of ion channel density as a target for cardiovascular disease. J Clin Invest.

[94] Rotsch C, Radmacher M (2000). Drug-induced changes of cytoskeletal structure and mechanics in fibroblasts: an atomic force microscopy study. Biophys J.

[95] Rubtsov AM, Lopina OD (2000). Ankyrins. FEBS Lett.

[96] Schwartz N, Hosford M, Sandoval RM, Wagner MC, Atkinson SJ, Bamburg J, Molitoris BA (1999). Ischemia activates actin depolymerizing factor: role in proximal tubule microvillar actin alterations. Am J Physiol.

[97] Shapovalov G, Lehen'kyi V, Skryma R, Prevarskaya N (2011). TRP channels in cell survival and cell death in normal and transformed cells. Cell Calcium.

[98] Simard JM, Tsymbalyuk O, Keledjian K, Ivanov A, Ivanova S, Gerzanich V (2012). Comparative effects of glibenclamide and riluzole in a rat model of severe cervical spinal cord injury. Exp Neurol.

[99] Simon F, Leiva-Salcedo E, Armisen R, Riveros A, Cerda O, Varela D, Eguiguren AL, Olivero P, Stutzin A (2010). Hydrogen peroxide removes TRPM4 current desensitization conferring increased vulnerability to necrotic cell death. J Biol Chem.

[100] Simon F, Varela D, Eguiguren AL, Diaz LF, Sala F, Stutzin A (2004). Hydroxyl radical activation of a Ca(2+)-sensitive nonselective cation channel involved in epithelial cell necrosis. Am J Physiol Cell Physiol.

[101] Sook HM, Shin KJ, Kim YH, Kim SH, Lee T, Kim E, Ho RS, Suh PG (2003). Thiram and ziram stimulate non-selective cation channel and induce apoptosis in PC12 cells. Neurotoxicology.

[102] Stallmeyer B, Zumhagen S, Denjoy I, Duthoit G, Hebert JL, Ferrer X, Maugenre S, Schmitz W, Kirchhefer U, Schulze-Bahr E, Guicheney P, Schulze-Bahr E (2012). Mutational spectrum in the Ca(2+)-activated cation channel gene TRPM4 in patients with cardiac conductance disturbances. Hum Mutat.

[103] Sudhandiran G, Shaha C (2003). Antimonial-induced increase in intracellular Ca^2+^ through non-selective cation channels in the host and the parasite is responsible for apoptosis of intracellular *Leishmania donovani* amastigotes. J Biol Chem.

[104] Tan HL, Fong WJ, Lee EH, Yap M, Choo A (2009). mAb 84, a cytotoxic antibody that kills undifferentiated human embryonic stem cells via oncosis. Stem Cells.

[105] Temkin V, Huang Q, Liu H, Osada H, Pope RM (2006). Inhibition of ADP/ATP exchange in receptor-interacting protein-mediated necrosis. Mol Cell Biol.

[106] Torgerson RR, McNiven MA (1998). The actin–myosin cytoskeleton mediates reversible agonist-induced membrane blebbing. J Cell Sci.

[107] Uchida K, Tominaga M (2011). The role of thermosensitive TRP (transient receptor potential) channels in insulin secretion. Endocr J.

[108] Ullrich ND, Voets T, Prenen J, Vennekens R, Talavera K, Droogmans G, Nilius B (2005). Comparison of functional properties of the Ca^2+^-activated cation channels TRPM4 and TRPM5 from mice. Cell Calcium.

[109] Vandenabeele P, Galluzzi L, Vanden Berghe T, Kroemer G (2010). Molecular mechanisms of necroptosis: an ordered cellular explosion. Nat Rev Mol Cell Biol.

[110] Vennekens R, Nilius B (2007) Insights into TRPM4 function, regulation and physiological role. Handb Exp Pharmacol:269–285 10.1007/978-3-540-34891-7_16 17217063

[111] Vennekens R, Olausson J, Meissner M, Bloch W, Mathar I, Philipp SE, Schmitz F, Weissgerber P, Nilius B, Flockerzi V, Freichel M (2007). Increased IgE-dependent mast cell activation and anaphylactic responses in mice lacking the calcium-activated nonselective cation channel TRPM4. Nat Immunol.

[112] Vereninov AA, Goryachaya TS, Moshkov AV, Vassilieva IO, Yurinskaya VE, Lang F, Rubashkin AA (2007). Analysis of the monovalent ion fluxes in U937 cells under the balanced ion distribution: recognition of ion transporters responsible for changes in cell ion and water balance during apoptosis. Cell Biol Int.

[113] Wagner MC, Molitoris BA (1997). ATP depletion alters myosin I beta cellular location in LLC-PK1 cells. Am J Physiol.

[114] White P, Doctor RB, Dahl RH, Chen J (2000). Coincident microvillar actin bundle disruption and perinuclear actin sequestration in anoxic proximal tubule. Am J Physiol Renal Physiol.

[115] White P, Gu L, Chen J (2002). Decreased actin solubility observed during ATP-depletion is mimicked by severing agents but not depolymerizing agents in isolated and cultured proximal tubular cells. Clin Physiol Funct Imaging.

[116] Xiao AY, Wei L, Xia S, Rothman S, Yu SP (2002). Ionic mechanism of ouabain-induced concurrent apoptosis and necrosis in individual cultured cortical neurons. J Neurosci.

[117] Ye X, Wang Y, Yang M, Wang Q, Liang Q, Ma Z, Zhang B, Gao Y (2009). Investigating the in vitro metabolism of veratridine: characterization of metabolites and involved cytochrome P450 isoforms. J Chromatogr B Analyt Technol Biomed Life Sci.

[118] Yu SP (2003). Na(+), K(+)-ATPase: the new face of an old player in pathogenesis and apoptotic/hybrid cell death. Biochem Pharmacol.

[119] Zhang T, Yong SL, Drinko JK, Popovic ZB, Shryock JC, Belardinelli L, Wang QK (2011). LQTS mutation N1325S in cardiac sodium channel gene SCN5A causes cardiomyocyte apoptosis, cardiac fibrosis and contractile dysfunction in mice. Int J Cardiol.

